# Strain-specific responses of avian influenza virus to disruption of solute carrier family 35 member A1 (SLC35A1) in chicken cells

**DOI:** 10.1016/j.psj.2026.107178

**Published:** 2026-05-25

**Authors:** Heidi Liu, Jéssica Moraes Cruvinel, Wesley C. Warren, Wenjun Ma, Paula R. Chen

**Affiliations:** a Division of Animal Sciences, University of Missouri, Columbia, MO 65211, USA; b USDA-ARS, Plant Genetics Research Unit, Columbia, MO 65211, USA; c Department of Pathobiology and Integrative Biomedical Sciences, University of Missouri, Columbia, MO 65211, USA; d Department of Molecular Microbiology and Immunology, University of Missouri, Columbia, MO 65211, USA; e NextGen Center for Influenza and Emerging Infectious Diseases, University of Missouri, Columbia, MO 65211, USA

**Keywords:** Avian influenza virus, CRISPR/Cas9, SLC35A1, Sialic acid, Genetic resistance

## Abstract

Avian influenza virus (AIV) poses a persistent threat to poultry health and food security, with conventional control measures offering limited protection. A promising alternative is the use of gene editing to generate host resistance by ablating viral entry receptors or cellular proteins that are required for completion of the viral life cycle. The solute carrier family 35 member A1 (*SLC35A1*) gene encodes a Golgi-localized CMP-sialic acid transporter that is a key step in the sialylation of glycoproteins. In this study, we used the CRISPR/Cas9 system to disrupt *SLC35A1* in chicken DF-1 fibroblasts and evaluated the effect on sialic acid expression and susceptibility to different strains of AIV. Lectin staining and flow cytometry confirmed a significant reduction in α2,3-linked sialic acids in *SLC35A1* knockout cells, while α2,6-linked sialic acids were absent in the cells regardless of genotype. Infection experiments with three avian influenza virus strains (H5N1/PR8, H5N2, and H7N1) revealed that *SLC35A1* knockout reduced viral replication in a strain-specific manner. Knockout cells infected with H5N1/PR8 showed the greatest dependence on SLC35A1-mediated sialylation with decreased viral load at 24 hours post-infection (hpi) and 48 hpi compared to wildtype cells and no observable viral growth between the timepoints. Infection of knockout cells with H5N2 resulted in a modest decrease in viral load at both timepoints as well as absence of viral growth. On the other hand, infection of knockout cells with H7N1 resulted in decreased viral load only at 48 hpi compared to wildtype cells, but the amount of virus in knockout cultures increased from 24 hpi to 48 hpi. These results demonstrate that SLC35A1 is a key host factor that supports AIV entry via α2,3-linked sialic acids; however, viral dependency on this host factor may be confounded by strain.

## Introduction

Reoccurring outbreaks of highly pathogenic avian influenza virus (AIV) continue to pose a significant challenge for the poultry industry with high mortality rates and major economic losses. Adherence to biosecurity measures to control the spread of highly pathogenic AIV within commercial poultry populations has shown limited success with the most recent outbreak in 2022 lasting several years with millions of birds lost. Therefore, alternative strategies are needed to protect critical commercial lines and reduce the risk of zoonoses. A promising approach to prevent AIV infection in poultry is through genome editing which has the potential to block infection at different stages in the viral life cycle and confer complete resistance over numerous generations (Chen et al., 2025).

Avian influenza virus binds to sialic acid residues on the host cell through its hemagglutinin (HA) protein to initiate entry. Sialic acids are nine-carbon molecules that are bound to galactose through α−2,3 or α−2,6 linkages with abundance varying based on tissue type. Typically, AIV strains demonstrate a preference for binding α−2,3 linked sialic acid residues, whereas human influenza virus strains generally bind α−2,6 linked sialic acids (Zhao and Pu, 2022). However, influenza A viruses (IAVs) can mutate to recognize both α−2,3 and α−2,6 linked sialic acids, thus expanding host range and transmissibility.

Genetic screens using the clustered regularly interspaced short palindromic repeats (CRISPR) technologies are a novel method of identifying host factors that are involved in pathogen infection. Multiple genome-wide screens have identified the solute carrier 35 member A1 (SLC35A1) as a key host factor that facilitates influenza virus infection (Chen, White, Walker, Kapczynski and Suarez, 2025), proving it to be a top candidate for genetic manipulation to confer resilience to AIV. The SLC35A1 gene encodes a cytidine 5′-monophosphate (CMP)-sialic acid transporter that is localized in the Golgi apparatus and is involved in the addition of sialic acids to the galactose residues of glycoproteins (Nishihara, 2014). To investigate the involvement of SLC35A1 in infection of different AIV strains, the *SLC35A1* gene was knocked out in DF-1 fibroblast cells that were infected with H5 or H7 AIV strains to assess susceptibility.

## Materials and methods

### Cell culture

Chicken embryonic fibroblast (DF-1; CRL-3586, ATCC, Manassas, VA) cells were grown in Dulbecco's Modified Eagle Medium (DMEM; Gibco, Waltham, MA) containing 10% fetal bovine serum (FBS; Cytiva, Marlborough, MA). Madin–Darby canine kidney (MDCK; CCL-34, ATCC) cells were cultured in minimum essential medium (MEM; Cytiva) supplemented with 10% FBS and 1% antibiotic-antimycotic solution (Anti-Anti; Gibco). All cells were cultured at 38°C in a humidified incubator with 5% CO_2_.

### Generation of SLC35A1 knockout cells

Two single guide RNAs (sgRNAs) were designed to target exon 3 (5′- AAATGTCTTTGGAAGTCCCAAGG) and exon 4 (5′- GCGGCGTTATACTTGTTCAGTGG) of the chicken *SLC35A1* gene by using CRISPOR (https://crispor.gi.ucsc.edu/) and were cloned into px330-mCherry which was a gift from Jinsong Li (Addgene plasmid #98750). DF-1 cells were transfected with 0.5 μg of plasmid DNA by using jetOPTIMUS transfection reagent (Sartorius AG, Göttingen, Germany) according to the manufacturer’s instructions. After 24 h of incubation, transfected cells were plated at a density of 100–200 cells per 10 cm culture dish to allow clonal expansion. Individual colonies were isolated after 10 days of culture and expanded in separate wells. Genomic DNA of *SLC35A1* for the colonies was amplified by PCR with forward primer 5′- CCTAGGATAGCCCTCAGTTAT and reverse primer 5′- ACTGGTTCCCAGCAAATG. PCR products of the knockout (KO) colonies were subjected to Sanger sequencing and TOPO cloning to confirm indels and knockout status. Four wildtype (WT) colonies were used as positive controls.

### Neuraminidase (NA) treatment

Neuraminidase was reconstituted in double-distilled water to a final concentration of 5 U/mL (MilliporeSigma, Burlington, MA) following the manufacturer’s instructions. For neuraminidase treatment, DF-1 cells in 24-well plate were washed two times with Dulbecco’s phosphate buffered saline (DPBS; Gibco) and then treated with NA at 10 mU/well in DMEM medium without FBS at 38°C, 5% CO_2_ for 2 h. After incubation, the cells were washed once with DPBS and cultured with DMEM medium with 10% FBS.

### Lectin staining

DF-1 cells (WT, NA-treated WT, and *SLC35A1* KO) were cultured without FBS for 2 h, fixed with 4% paraformaldehyde for 15 min at room temperature, permeabilized with 0.1% Triton X-100, and blocked with Carbo-Free Blocking Solution (#SP-5040-125, Vector Laboratories, Newark, CA) for 1 h. Cells were incubated with biotinylated Sambucus nigra lectin (SNA, #B-1305, Vector Laboratories) or biotinylated Maackia amurensis lectin II (MALII, #B-1265, Vector Laboratories) at 1:500 dilution for 1 h at room temperature. After washing, DyLight 649-conjugated streptavidin (#SA-5649-1, Vector Laboratories) was added for 30 min. Nuclei were stained with DAPI, and cells were imaged by using a Nikon Ti2‑E widefield fluorescence microscope equipped with a 40 × /1.30 NA oil‑immersion objective. DyLight 649 (Cy5‑equivalent) fluorescence was excited and emitted through the appropriate far‑red filter set. Images were captured using a Sona sCMOS camera (Andor) operating in high‑quality mode with 85 ms exposure time for the 640‑nm channel and 16‑bit high‑dynamic‑range acquisition. Three‑dimensional image stacks were acquired using the Z‑scan protocol with a 1 µm step size across 21 optical sections spanning from 2.9 µm to 17.6 µm. Raw image files were imported into Imaris 10.2.0 (Oxford Instruments) for visualization. Signal intensity filters were applied uniformly to all datasets with threshold values set to minimum = 120 and maximum = 10,000 to enhance true fluorescent signal while suppressing background noise. Final renderings were generated using standard Imaris volume‑rendering settings without additional deconvolution.

### Flow cytometry

Collected cells were washed twice with DPBS and blocked with Carbo-Free blocking solution (SP-5040-125, Vector Laboratories) for 30 min on ice. Suspensions were incubated with biotinylated lectins (SNA or MAL II, 1:500 in DPBS) for 30 min on ice, followed by FITC-conjugated streptavidin (#SA-5001-1, Vector Laboratories) for 15 min on ice. After washing and filtering through a 70-µm strainer, samples were analyzed on Cytek Aurora full-spectrum flow cytometer (Aurora; Cytek Biosciences, Fremont, CA). Controls included unstained cells, secondary-only staining, and neuraminidase-treated samples. Flow cytometry data were analyzed using FlowJo v10. Live singlet cells were gated, and lectin binding was quantified as mean fluorescence intensity (MFI) in the FITC channel. Background fluorescence from neuraminidase-treated controls was subtracted. Corrected MFI values were normalized to WT control colonies, which were set to 100%.

### Viruses

Influenza A viruses, including rg-A/American wigeon/South Carolina/22-000345-001/2021 (rgH5N1/PR8), A/Shorebird/DE/518/2021 (H5N2), and A/NOPI/ALB/77/2012 (H7N1), were kindly provided by Dr. Richard Webby from St. Jude Children's Research Hospital and used in this study. The rgH5N1/PR8 virus is a reverse genetics-derived virus that contains six internal genes from the PR8 H1N1 virus and surface NA and modified HA genes from the clade 2.3.4.4b A/American wigeon/South Carolina/22-000345-001/2021 (H5N1) virus. These three viruses were propagated in the allantoic cavity of 9-day-old embryonated chicken eggs. Allantoic fluid was pooled from multiple eggs, clarified by centrifugation, aliquoted and frozen at −80°C.

The tissue culture infectious dose 50% (TCID₅₀) was used to determine the titers of amplified influenza viruses on MDCK cells. Virus samples were serially diluted 10-fold in infection medium (MEM supplemented with 2% FBS and 1 μg/mL TPCK-treated trypsin). Each dilution was added to 4 replicate wells (100 μL per well) and incubated at 37°C for 72 hours. The number of positive wells at each dilution was recorded, and the TCID₅₀/mL was calculated by using the Reed–Muench method.

### Influenza virus infection

Cells were grown to 90-100% monolayer confluence in 48-well plates and then infected with the specified virus in triplicate at a multiplicity of infection of 0.01 for 1 h with 15 min interval shaking. The unattached virus particles were washed and replaced with 200 ul DMEM and then incubated at 38°C for 48 h. The supernatants were collected at 24 hours post-infection (hpi) and 48 hpi for RT-PCR analysis.

### Reverse transcription quantitative PCR (RT-qPCR)

Total RNA from WT or KO colonies was extracted by using the RNeasy Mini Kit according to the manufacturer’s instructions (Qiagen, Germantown, MD). Synthesis of cDNA was performed by using the SuperScript IV VILO Master Mix according to the manufacturer’s instructions (Thermo Fisher). The primer sequence for *SLC35A1* (NM_204513.2) was F: 5`- CGGGATTTGCAGGAGTTTAT and R: 5`- GTACACGCCAACCAAAGT, size: 116 bp). Beta-actin (*ACTB*, NM_205518.2 F: 5`- GCCAACAGAGAGAAGATGAC, R: 5`-CACCAGAGTCCATCACAATAC, size: 130 bp) was used as a housekeeping gene. After diluting cDNA samples to 5 ng/μL, reactions were run in triplicate on a QIAquant 96 5plex (Qiagen) with the conditions 95°C for 3 min, and 35 cycles of 95°C for 10 s, 55°C for 10 s, and 72°C for 30 s. Afterwards, a dissociation curve was generated to ensure that a single product was amplified. The abundance of each transcript was calculated relative to *ACTB*, which was not statistically different between groups (*P* > 0.05), and then normalized to the abundance of the WT colonies. The comparative threshold cycle method (2^-ΔΔCt^) was used to determine transcript abundance for each sample.

To detect viral RNA from medium samples, RT-qPCR assays were performed using the CDC-developed assay targeting the influenza A M gene (Selvaraju and Selvarangan, 2010). The primers were forward 5′-GACCRATCCTGTCACCTCTGAC-3′ and reverse 5′-AGGGCATTYTGGACAAAKCGTCTA-3′. The probe was 5′- (FAM)-TGCAGTCCTCGCTCACTGGGCACG-(BHQ1)-3′. RNA was extracted by using the QIAamp Viral RNA Mini Kit (Qiagen, Germantown, Maryland). Then, RT-qPCR was performed in triplicate by using the qScript XLT 1-Step RT-qPCR ToughMix (Quantabio, Beverly, Massachusetts) according to the manufacturer’s instructions on the CFX384 Touch Real-Time PCR Detection System (Bio-Rad, Hercules, CA). The PCR program consisted of reverse transcription for 10 minutes at 50°C followed by 1 minute at 95°C, 40 cycles of 95°C for 10 seconds, and 60°C for 60 seconds. A cutoff Ct value of 36 or lower was considered positive.

### Statistical analysis

All experiments were repeated at least three times so that replicate variation could be assessed. Analyses of quantitative data were performed by using SAS software, version 9.4 (SAS Institute Inc., Cary, NC). The Shapiro–Wilk test was used for assessing the normality assumption for each experiment, and data were log transformed if deviation from the normality assumption was detected. Data were analyzed by using a two-way ANOVA with genotype and timepoint as fixed factors, and culture as the experimental unit. If significant effects were observed after the ANOVA, the means were compared by Tukey's honest significant difference (HSD) multiple comparison test. Analysis of data consisting of two groups was performed by using Student’s t-test. Differences were considered significant at *P* < 0.05.

## Results and discussion

Numerous CRISPR screens have been conducted to identify host factors that are required by IAVs to complete their life cycle, and SLC35A1 has been identified as a potentially important factor across species by transporting sialic acids that are necessary for viral entry (Chen, White, Walker, Kapczynski and Suarez, 2025). However, most studies only use one influenza virus strain to perform the screens, resulting in uncertainty whether the identified host factors are ubiquitously required. The objective of this study was to introduce edits into the chicken *SLC35A1* gene to ablate function and assess infection and replication of different H5 and H7 AIVs.

**Fig. 1:**
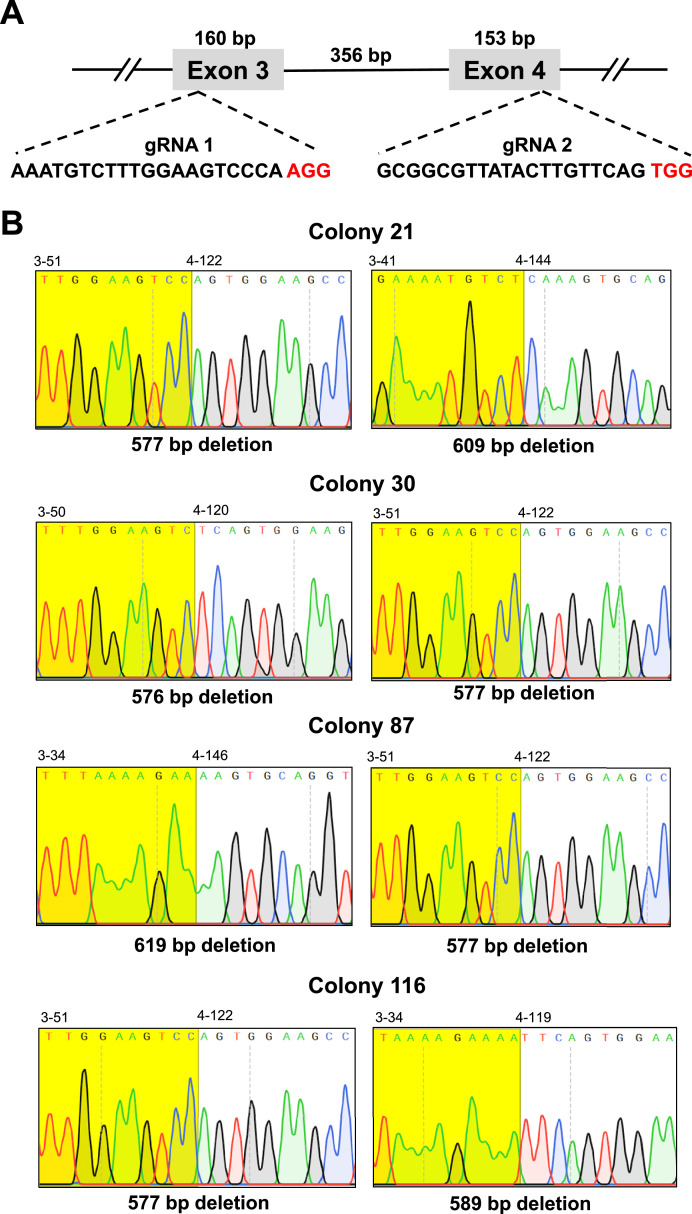
**Generation of *SLC35A1* knockout chicken cells. (A)** Schematic depicting the region of the *SLC35A1* gene targeted for editing with the CRISPR/Cas9 system. Guide RNA sequences are shown under dashed lines with PAM sequences in red. **(B)** Chromatograms of edited colonies that were selected for downstream analyses. Yellow highlighted bases indicate upstream region of the edit, and unhighlighted bases indicate downstream region of the edit. Notation of #-# above the chromatograms represents the exon number followed by the exact nucleotide position within the exon.

Chicken *SLC35A1* is located on chromosome 3 (NC_052534.1) and contains 8 exons. Due to a possible splicing variant, sgRNAs were designed in exons 3 and 4 to introduce frameshift mutations in order to ablate the transporter function of the protein (Fig. 1**A**). Numerous colonies with different *SLC35A1*-edited alleles were generated and screened as biological replicates. Four *SLC35A1*-edited colonies (#21, 30, 87, 116) demonstrated deletions spanning the two sgRNAs of varying sizes and were selected for further analyses. The genotypes of these colonies are illustrated in Fig. 1**B**. Relative *SLC35A1* transcript abundance measured by qPCR showed a decrease in the KO colonies (0.27 ± 0.06) compared to WT colonies (1.0 ± 0.05, *P* < 0.001).

**Fig. 2:**
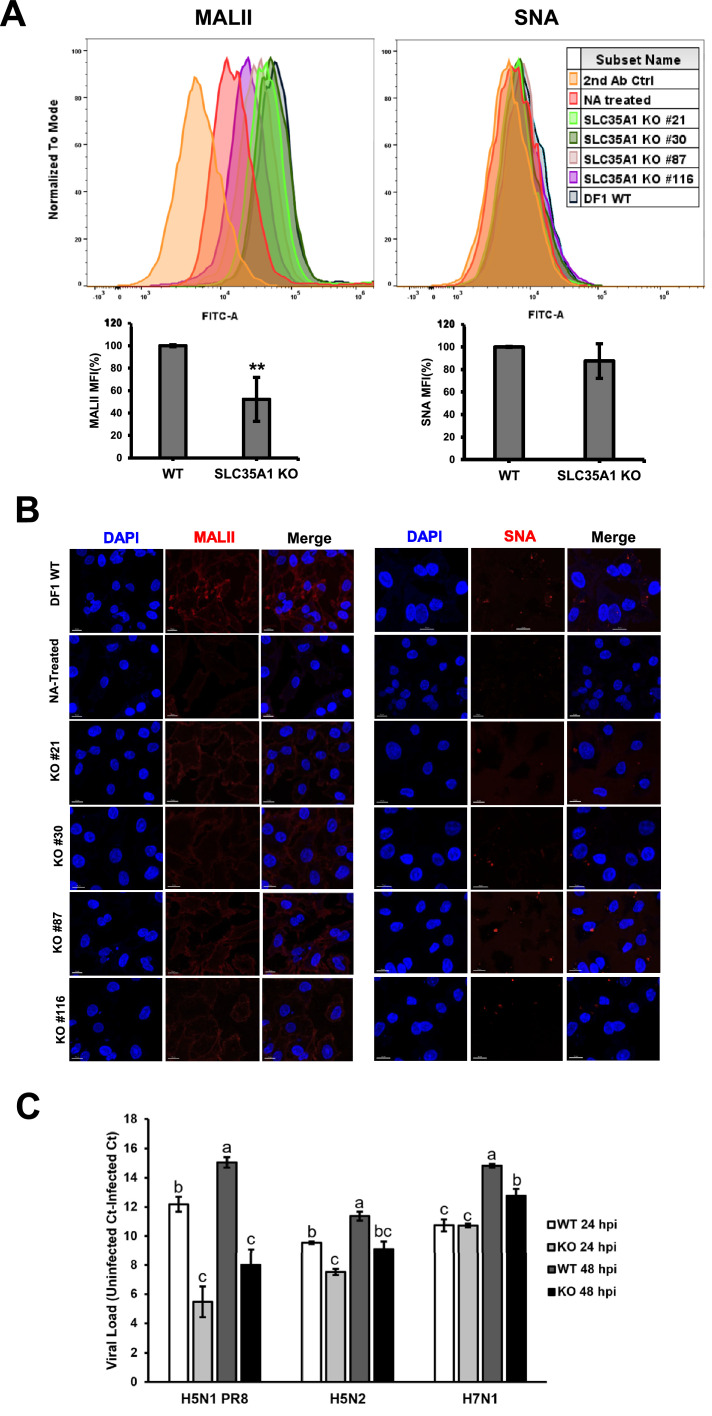
**Chacterization of sialic acid expression and infection by avian influenza virus strains. (A)** Representative flow cytometry histrograms after staining WT and KO cells with MALII to detect α−2,3 linked sialic acids or SNA to detect α−2,6 linked sialic acids on the cell surface. Neuraminadase (NA) treatment to remove surface sialic acids and staining with the secondary antibody only were performed as controls. Mean fluorescence intensity (MFI) was quantified across three replicates, and data represent mean ± SEM from four WT or KO colonies. ** above the bars indicates *P* < 0.01. **(B)** Representative images of WT, NA-treated, and KO cells after staining with MALII and SNA to detect surface sialic acids. Scale bars equal 10 μm. **(C)** Viral load in the medium assessed by RT-qPCR after infection with H5N1 PR8, H5N2, or H7N1 at 24 hpi or 48 hpi. Infected samples were normalized to uninfected samples, and three replicates were assessed. Data are presented as means ± SEM from four WT or KO colonies. Different lowercase letters above the bars represent statistical differences between groups (*P* < 0.05).

As previously mentioned, sialic acid residues are known to mediate entry of influenza viruses in host cells. Normally, AIV strains prefer to bind to α2,3-linked sialic acids, while human influenza virus strains prefer α2,6-linked sialic acids (Zhao and Pu, 2022). Various lectins have been shown to bind sialic acids based on linkage specificity. For example, Maackia amurensis lectin II (MALII) has been used for detection of α2,3-linked sialic acids, while Sambucus nigra lectin (SNA) has been employed for detecting α2,6-linked sialic acids in different cell types (Ma, et al., 2023; Nelli, et al., 2024; Zhao and Pu, 2022). To determine the impact of the *SLC35A1* edits on sialic acid expression, MALII and SNA staining was performed to detect α2,3- and α2,6-linked sialic acids, respectively, in WT and KO DF-1 colonies by using flow cytometry and confocal microscopy. Treatment with neuraminidase (NA) completely abolished sialic acid expression in WT cells, confirming the specificity of lectin binding. A marked reduction of α2,3-linked sialic acids in *SLC35A1* KO colonies was observed compared to WT cells as indicated by decreased MALII binding and MFI (Fig. 2**A**, MALII panel, *P* < 0.01); however, the presence of a weak MALII signal in these cells may be due to other compensatory sialic acid transport mechanisms. For instance, *de novo* synthesis pathways may be upregulated, or other nucleotide-sugar transporters, including solute carrier family 35 member A2 (SLC35A2), could mediate transport (Ma, et al., 2021). In contrast, α2,6-linked sialic acids were not detected in both WT and KO cells as all histograms, including negative controls, overlapped (Fig. 2**A**, SNA panel). These findings were further confirmed by confocal microscopy where MALII staining revealed substantially weaker signals in KO cells compared to WT cells, consistent with reduced terminal galactose residues (Fig. 2**B**). Conversely, SNA staining produced only background signals across all groups, indicating minimal or no α2,6-linked sialic acid expression in DF-1 cells. Overall, these results demonstrate that α2,3-linked sialic acids are the predominant form in DF-1 cells and that mutations in *SLC35A1* significantly diminish their surface expression.

The hemagglutinin (HA) glycoprotein of IAVs binds to sialic acid-containing glycans on the host cell surface, triggering clathrin-mediated endocytosis and subsequent internalization. Therefore, SLC35A1 is critical for maintaining the cellular sialylation landscape that supports HA-mediated binding and infection. Disruption of SLC35A1 function results in a marked reduction of cell surface sialic acids, which impairs both HA-receptor engagement and viral entry (Suzuki, 2005). However, the magnitude of this effect may vary among different AIV strains due to differences in HA-receptor interactions. To evaluate the contribution of SLC35A1 to AIV infection and replication, we infected WT and *SLC35A1* KO DF-1 colonies with three AIV strains (H5N1/PR8, H5N2, and H7N1) at an MOI of 0.01. Viral load in the medium of infected cells was quantified by RT-qPCR at 24 hpi and 48 hpi as an indicator of viral growth and replication (Fig. 2**C**).

Dependency upon SLC35A1 varied markedly among the three virus strains tested (Fig. 2**C**). Wildtype cells supported robust H5N1/PR8 viral replication with viral load values increasing from 24 hpi to 48 hpi while *SLC35A1* KO cells exhibited substantially reduced viral load at both 24 hpi and 48 hpi compared to WT cells (*P* < 0.001). Noticeably, no growth between the timepoints was observed in the KO cells, indicating strong dependence on SLC35A1-mediated sialylation for this strain. In contrast, the H5N2 virus demonstrated intermediate SLC35A1 dependency. WT cells showed increases in H5N2 viral load from 24 hpi to 48 hpi (*P* < 0.05), while no difference in viral load in KO cells was observed between these two timepoints. However, KO cells showed decreases in viral load compared to WT cells at both 24 hpi and 48 hpi (*P* < 0.05). An increase in viral load of H7N1 infected WT or KO cells was observed from 24 hpi to 48 hpi (*P* < 0.05), which was not observed in both H5 strains. Interestingly, H7N1 viral load was not reduced in *SLC35A1* KO cells compared to WT cells at 24 hpi, but a lower viral load was noted at 48 hpi when compared to WT cells, suggesting that H7N1 may compensate for reduced sialic acid availability during initial infection cycles, with a more noticeable effect after multi-cycle replication.

These results in this study demonstrate that CRISPR/Cas9-mediated editing of *SLC35A1* in chicken cells reduces replication of AIVs in a strain-specific manner, which may reflect variations in HA binding affinity for α2,3-linked sialic acids. Therefore, direct receptor-binding studies are needed to confirm this relationship. H5N1/PR8, which exhibits a strong dependency for binding to α2,3-linked sialic acids, was most severely impacted by SLC35A1 disruption. H5N2 showed intermediate sensitivity to *SLC35A1* KO, consistent with variable HA binding properties among H5 subtypes (Weber, et al., 2026). In contrast, H7N1 displayed only modest sensitivity, suggesting that this strain may either interact with sialic acids through lower affinity or utilize alternative receptors or entry pathways during early infection. The dependency of this strain on SLC35A1-mediated sialylation may only be evident during multi-cycle replication.

The observed strain-specific differences likely reflect variations in HA binding affinity for α2,3-linked sialic acids, spatial distribution of receptors on the cell surface, or the capacity to employ alternative co-receptors or entry mechanisms (Adu, et al., 2025). These findings underscore the complexity of AIV-host interactions and indicate that targeting sialylation pathways, such as SLC35A1, could provide differential antiviral protection depending on the circulating viral strain (Kamiki, et al., 2022). Further studies using additional virus strains and receptor-binding analyses are necessary to elucidate the underlying mechanisms.

## CRediT authorship contribution statement

**Heidi Liu:** Writing – review & editing, Writing – original draft, Visualization, Validation, Methodology, Formal analysis, Data curation, Conceptualization. **Jéssica Moraes Cruvinel:** Writing – review & editing, Data curation. **Wesley C. Warren:** Writing – review & editing, Supervision, Resources. **Wenjun Ma:** Writing – review & editing, Supervision, Resources. **Paula R. Chen:** Writing – review & editing, Writing – original draft, Visualization, Validation, Supervision, Resources, Project administration, Methodology, Investigation, Funding acquisition, Formal analysis, Conceptualization.

## Disclosures

The authors declare that they have no competing interests.

## References

[bib0001] Adu O.F., Sempere Borau M., Früh S.P., Karakus U., Weichert W.S., Wasik B.R., Stertz S., Parrish C.R. (2025). Cell binding, uptake, and infection of influenza A virus using recombinant antibody-based receptors. J. Virol..

[bib0002] Chen P.R., White S.N., Walker L.R., Kapczynski D.R., Suarez D.L. (2025). Genetic resilience or resistance in poultry against avian influenza virus: mirage or reality?. J. Virol..

[bib0003] Kamiki H., Murakami S., Nishikaze T., Hiono T., Igarashi M., Furuse Y., Matsugo H., Ishida H., Katayama M., Sekine W., Muraki Y., Takahashi M., Takenaka-Uema A., Horimoto T. (2022). Influenza A virus agnostic receptor tropism revealed using a novel biological system with terminal sialic acid knockout cells. J. Virol..

[bib0004] Ma T., Niu S., Wu Z., Pan S., Wang C., Shi X., Yan M., Xu B., Liu X., Li L., Yan D., Teng Q., Yuan C., Pan X., Zhang Z., Duc H.M., Li Z., Liu Q. (2023). Genome-wide CRISPR screen identifies GNE as a key host factor that promotes influenza A virus adsorption and endocytosis. Microbiol. Spect..

[bib0005] Ma X., Li Y., Kondo Y., Shi H., Han J., Jiang Y., Bai X., Archer-Hartmann S.A., Azadi P., Ruan C., Fu J., Xia L. (2021). Slc35a1 deficiency causes thrombocytopenia due to impaired megakaryocytopoiesis and excessive platelet clearance in the liver. Haematologica.

[bib0006] Nelli R.K., Harm T.A., Siepker C., Groeltz-Thrush J.M., Jones B., Twu N.C., Nenninger A.S., Magstadt D.R., Burrough E.R., Piñeyro P.E., Mainenti M., Carnaccini S., Plummer P.J., Bell T.M. (2024). Sialic acid receptor specificity in mammary gland of dairy cattle infected with highly pathogenic Avian Influenza A(H5N1) virus. Emerg. Infect. Dis..

[bib0007] Nishihara S., Taniguchi N., Honke K., Fukuda M., Narimatsu H., Yamaguchi Y., Angata T. (2014). Handbook of Glycosyltransferases and Related Genes.

[bib0008] Selvaraju S.B., Selvarangan R. (2010). Evaluation of three influenza A and B real-time reverse transcription-PCR assays and a new 2009 H1N1 assay for detection of influenza viruses. J. Clin. Microbiol..

[bib0009] Suzuki Y. (2005). Sialobiology of influenza: molecular mechanism of host range variation of influenza viruses. Biol. Pharm. Bull..

[bib0010] Weber J., Ponse N.L.D., Zhu X., Ríos Carrasco M., Han A.X., Funk M., Lin T.-H., Gabarroca García A., Spruit C.M., Zhang D., Yu W., Wilson I.A., Richard M., Boons G.-J., de Vries R.P. (2026). The receptor binding properties of H5Ny influenza A viruses have evolved to bind to avian-type mucin-like O-glycans. PLOS Pathog..

[bib0011] Zhao C., Pu J. (2022). Influence of host sialic acid receptors structure on the host specificity of influenza viruses. Viruses.

